# Orbital Apex Involvement in Sinonasal Intestinal-Type Adenocarcinoma: Impact on Distant Metastasis and Mortality

**DOI:** 10.1055/s-0046-1825833

**Published:** 2026-10-14

**Authors:** António Andrade, José Maria Sousa, José Alberto Fernandes, Ricardo Vaz, Manuel Mendes Leal, Gil Coutinho

**Affiliations:** 1 Department of OtorhinolaryngologyUnidade Local de Saúde São JoãoPortoPortugal; 2 Unit of OtorhinolaryngologyDepartment of Surgery and PhysiologyFaculty of Medicine, Universidade do PortoPortoPortugal; 3 Department of NeuroradiologyUnidade Local de Saúde São JoãoPortoPortugal; 4 Center for Research on Health Technology and Services/Health Research Network (CINTESIS@RISE), Faculty of Medicine, Universidade do PortoPortoPortugal; 5 Department of OtorhinolaryngologyUnidade Local de Saúde de Trás-os-Montes e Alto DouroVila RealPortugal; 6 Department of BiomedicineUnit of Anatomy, Faculty of Medicine, Universidade do PortoPortoPortugal; 7 NeuroGen Research Group, Center for Research on Health Technology and Services (CINTESIS), Faculty of Medicine, Universidade do PortoPortoPortugal; 8 Department of OtorhinolaryngologyHospital Companhia União Fabril (CUF) TrindadePortoPortugal

**Keywords:** intestinal-type adenocarcinoma, paranasal sinus neoplasms, orbital apex, neoplasm metastasis, exenteration

## Abstract

**Introduction:**

Sinonasal intestinal-type adenocarcinoma (ITAC) is often diagnosed at advanced stages. Prognosis is strongly influenced by tumor extension into critical anatomical regions contiguous with the orbit and skull base, where achieving negative margins is challenging.

**Objective:**

To identify predictors of distant metastasis and disease-specific mortality in patients with ITAC, focusing on involvement of critical anatomical structures.

**Methods:**

Retrospective analysis of ITAC cases diagnosed between 2010 and 2024 at a tertiary center. Data on clinical presentation, imaging, histopathology, treatment modalities, and outcomes were collected. Uni- and multivariate analyses were performed to identify predictors of distant metastasis and disease-specific mortality.

**Results:**

Thirty-four ITAC cases were included. Distant metastasis occurred in 21.2% of cases; orbital apex ( *p* = 0.007) and pterygopalatine fossa ( *p* = 0.034) involvement were significant in univariate analysis, with only orbital apex remaining significant in multivariate analysis ( *p* = 0.024). Regarding ITAC-specific mortality, orbital apex involvement ( *p* = 0.002), relapse ( *p* = 0.043), and adjuvant radiotherapy ( *p* = 0.045) were associated factors, with radiotherapy showing a protective effect. Multivariate analysis confirmed orbital apex as a predictor of mortality ( *p* = 0.002), while adjuvant radiotherapy had a borderline association ( *p* = 0.050).

**Conclusion:**

Orbital apex involvement independently signals high risk of distant metastasis and disease-specific death in ITAC; such patients merit individualized, intensified multimodal management. Adjuvant radiotherapy may confer a protective benefit, particularly in advanced-stage disease.

## Introduction

Sinonasal neoplasms, although rare, are linked to significant morbidity and mortality, and intestinal-type adenocarcinoma (ITAC) is the most common non-squamous epithelial neoplasm, which is strongly linked with occupational exposure to wood and leather dust, a key risk factor for disease development. 1 2 Histologically, ITAC resembles adenocarcinomas of the gastrointestinal tract and is classified into five main subtypes: papillary, colonic, solid, mucinous, and mixed. 3 Histological differentiation is important in prognosis, with well-differentiated tumors tending to show better clinical outcomes compared to those of higher grade. 4

The high morbidity and mortality associated with ITAC are partially due to its insidious onset and nonspecific symptoms, such as nasal obstruction, rhinorrhea, and epistaxis, often leading to delayed diagnosis. The location of these tumors in pneumatized regions, such as the paranasal sinuses, allows for asymptomatic growth until advanced stages. 5 Neurological or ophthalmological symptoms usually indicate greater disease extension and are linked to reduced survival. 6 Tumor removal is challenging due to proximity to vital structures such as the orbit and intracranial contents. Local recurrence is common, occurring in up to 50% of cases, particularly when positive surgical margins are present or when the tumor extends into adjacent critical structures, such as the dura mater or orbit. 7

Although the primary characteristic of ITAC is its local aggressiveness, distant metastatic spread, while less frequent, does occur and is more common than regional lymph node dissemination. Distant metastases typically involve the lungs, liver, bones, and, more rarely, the central nervous system. This tumor spreading may develop several years after the initial treatment, highlighting the importance of long-term follow-up. 5

Given the severity of involvement of critical anatomical structures and ITAC's distinct clinicopathologic characteristics, the present study aims to characterize an ITAC restricted cohort and to quantify the association between extension to key areas and distant metastatic spread and mortality.

## Methods

A retrospective observational study was conducted at the Department of Otorhinolaryngology in a tertiary hospital between January 2010 and June 2024. Patients diagnosed with ITAC were included, with those lost to follow-up excluded from the analysis. Data were retrieved from medical records, including age, sex, initial symptoms, physical examination findings, medical history, and risk factors such as occupational exposure to wood dust. Treatment types were evaluated, and for patients undergoing surgery, the surgical approach was documented. Histopathological evaluation was carried out, including histological classification according to Barnes. 3

Imaging studies were reviewed by one of the authors (JMS) and the affected regions were catalogued in several areas: maxillary, frontal, and sphenoidal sinuses, orbit, orbital apex, cavernous sinus, extradural space, anterior and middle cranial fossa, pterygopalatine fossa, parapharyngeal space, and skin. In cases in which images were unavailable, the affected regions were identified using available reports. Tumors were restaged according to the 8th edition of the American Joint Committee on Cancer (AJCC) classification. 8 The study evaluated persistent or recurrent neoplasia, nodal and distant metastasis, and mortality during the follow-up period. Predictive factors for distant metastasis and death related to ITAC were evaluated, including age, areas involved by the tumor, histological subtypes, exenteration, positive margins, and adjuvant radiotherapy. Outcomes, including 5-year overall survival (5-OS) and 5-year disease-specific survival (5-DSS), were calculated using the Kaplan-Meier method.

Statistical analysis was conducted using IBM SPSS Statistics for Windows (IBM Corp.), version 27.0, with statistical significance defined as *p* < 0.05. Categorical variables were summarized by absolute and relative frequencies. The Kolmogorov-Smirnov test determined the normality of continuous variables. Continuous variables with normal distributions reported using the mean and standard deviation, while non-normally distributed variables were presented using the median and interquartile range. Survival outcomes were estimated with the Kaplan-Meier method, and multivariable prognostic factors were analyzed using Cox proportional hazards regression.

## Results

Thirty-four cases of ITAC in the sinonasal region were diagnosed, with no patients lost to follow-up. The median follow-up duration was 33.5 (40.3) months. All patients were male, with a mean age of 60 (± 11) years. Chronic exposure to wood dust was reported in 29 cases (85.3%). Symptoms and clinical findings are detailed in Table 1 . Ocular symptoms occurred in 9 cases (26.5%). Table 2 summarizes the complementary diagnostic exam data. Imaging was unavailable in two cases for assessment of tumor extent. The maxillary sinus, sphenoid sinus, and orbit were the anatomical regions with the highest frequency of tumor involvement. Papillary, mucinous, and colonic subtypes were the predominant histopathological classifications identified in the cohort. The degree of differentiation was not specified in 18 cases (52.9%). Moderate differentiation was observed in 14 cases (41.2%), while well-differentiated tumors were present in two cases (5.9%). A total of 30 cases (88.3%) were diagnosed at advanced stages (stage III or IV), reflecting a predominance of late-stage presentation. One patient presented with distant metastasis at the time of diagnosis and was referred for palliative management. Surgical treatment was performed in 33 patients (97.1%), including 19 cases (55.9%) managed with sinonasal surgery alone and 14 cases (41.2%) with combined sinonasal and neurosurgical approaches. Two patients (5.9%) underwent orbital exenteration. Adjuvant radiotherapy was administered in 25 cases (73.5%). During follow-up, distant metastasis occurred in 7 cases (21.2%), while nodal metastasis was observed in only 1 case (3%).

**Table 1:** Patients' symptoms and signs

	N (%)
Nasal obstruction	22 (64.7)
Epistaxis	17 (50)
Headache	10 (29.4)
Ocular symptoms and signs	9 (26.5)
Proptosis	6 (17.6)
Diplopia	5 (14.7)
Facial pain	5 (14.7)
Rhinorrhea	4 (11.8)

**Table 2:** Imaging and histopathology data

Extension of the lesion	N (%)
Maxillary sinus	26 (78.8)
Sphenoid sinus	23 (69.7)
Orbit	17 (51.5)
Frontal sinus	16 (50)
Anterior cranial fossa	13 (39.4)
Orbital apex	11 (33.3)
Extradural	9 (28.1)
Pterygopalatine fossa	8 (23.5)
Middle cranial fossa	7 (21.9)
Cavernous sinus	5 (15.6)
Parapharyngeal space	1 (3.1)
Skin	1 (2.9)
**Staging (AJCC 8th edition)**	
I	1 (2.9)
II	3 (8.8)
III	4 (11.8)
IV	26 (76.5)
**ITAC histopathology classification**	
Papillary	12 (35.3)
Mucinous	11 (32.4)
Colonic	5 (14.7)
Mixed	3 (8.8)
Solid	1 (2.9)
NOS	2 (5.9)

**Abbreviations** : AJCC, American Joint Committee on Cancer; ITAC, intestinal-type adenocarcinoma; NOS, not otherwise specified.

Total mortality reached 16 cases (47.1%), including one death due to cardiac pathology unrelated to the sinonasal neoplasia. The 5-year overall survival rate was 43.9%, with a 5-year disease-specific survival rate of 45.8%. These data are presented in Table 3 and Figs. 1 2 .

**Fig. 1:**
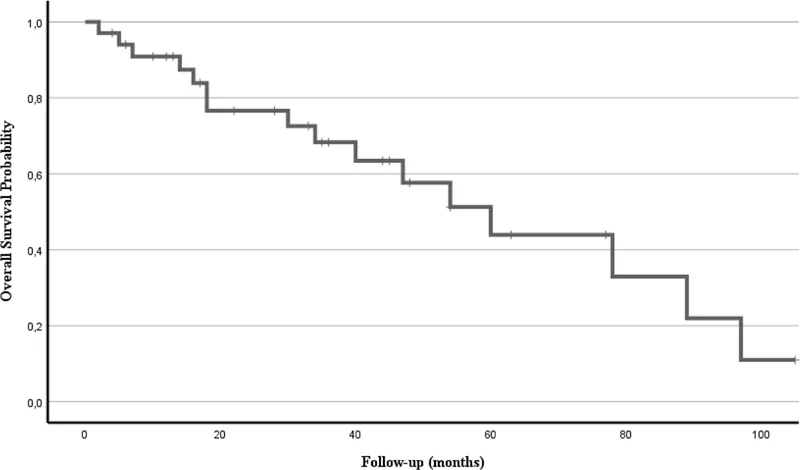
Kaplan-Meier curve for overall survival in patients with intestinal-type adenocarcinoma.

**Fig. 2:**
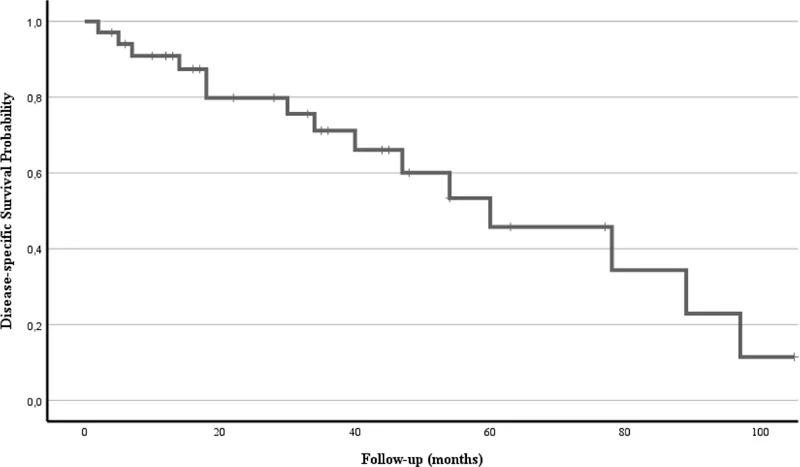
Kaplan-Meier curve for disease-specific survival in patients with intestinal-type adenocarcinoma.

**Table 3:** Treatment and follow-up variables

Treatment	N (%)
Isolated sinonasal surgery	19 (55.9)
Cranio-endoscopic resection	14 (41.2)
Orbital exenteration	2 (5.9)
Adjuvant radiotherapy	25 (73.5)
Palliative care	1 (2.9)
Positive margins	10 (30.3)
Relapse	11 (33.3)
Developed distant metastasis	7 (21.2)
**Mortality**	
Overall	16 (47.1)
Directly related to intestinal-type adenocarcinoma	15 (44.1)

Predictive factors analyzed included age, anatomical areas involved, histological subtype, orbital exenteration, surgical margins, adjuvant radiotherapy and relapse. Concerning the risk of distant metastasis development, the univariate analysis showed a significant association both with orbital apex involvement ( *p* = 0.007) and with pterygopalatine fossa spreading ( *p* = 0.034). However, in the multivariate analysis, only orbital apex involvement remained significantly associated with distant metastasis ( *p* = 0.024) ( Table 4 ). Regarding ITAC-specific mortality, univariate analysis identified orbital apex involvement ( *p* = 0.002), relapse ( *p* = 0.043), and adjuvant radiotherapy ( *p* = 0.045) as significant factors, with adjuvant radiotherapy demonstrating a protective effect. In the multivariate model, orbital apex involvement remained significantly associated with ITAC-related mortality ( *p* = 0.002), while adjuvant radiotherapy showed a borderline association ( *p* = 0.050) ( Table 5 ).

**Table 4:** Univariate and multivariate analysis for factors associated with progression to distant metastasis

	Univariate			Multivariate		
	Hazard ratio	*p* -value	95%CI	Hazard ratio	*p* -value	95%CI
Orbital apex	25.200	**0.007**	2.450–259.228	22.400	**0.024**	1.495–335.519
Pterygopalatine fossa	7.333	**0.034**	1.168–46.052	1.223	0.868	0.114–13.150

**Table 5:** Univariate and multivariate analysis for factors associated with death directly related to intestinal-type adenocarcinoma

	Univariate			Multivariate		
	Hazard ratio	*p* -value	95%CI	Hazard ratio	*p* -value	95%CI
Orbital apex	34	**0.002**	3.461–333.974	109.158	**0.002**	5.22–221.386
Relapse	5.333	**0.043**	1.058–26.898	0.942	0.968	0.050–17.739
Adjuvant radiotherapy	0.157	**0.045**	0.026–0.956	0.031	0.050	0.001–0.996

## Discussion

Sinonasal ITACs are associated with exposure factors such as wood dust. 1 2 In Portugal, this pathology is more frequently diagnosed due to the presence of the wood industry, particularly involving oak, beech, and cork. 2 9 In our sample, 85.3% of patients were exposed to this risk factor, although this percentage could be higher, as a detailed and thorough history is often required to accurately determine lifetime exposure. 10 The predominance of male patients is linked to occupational exposure, as shown in several studies. 3 5 11 This risk factor is crucial for distinguishing occupational adenocarcinoma from sporadic ITAC. One reason is the possibility of multiple adenocarcinoma foci in regions exposed to occupational risk factors. 9

Adenocarcinomas commonly arise in the ethmoidal region and have the potential to develop in the contralateral ethmoid sinus, even when initially unaffected. To address this risk, some authors recommend bilateral ethmoidectomy as a proactive surgical strategy to reduce recurrence and enhance disease management. 1 In our cohort, unilateral resection was preferred whenever oncologically safe as we believe it provides an appropriate surgical management for selected unilateral ITAC. In this regard, Schreiber and coworkers concluded that selected unilateral resection presented similar survival rates and lower morbidity compared to bilateral surgery. 12 Moreover, and as expected, it was also found the unilateral surgery was associated with greater olfactory function. Additionally, the latency period until the appearance of this carcinoma can be significant, even with short exposure times, such as 2 years. 4 10 This latency can extend to over 60 years after exposure. 4 10 13 Therefore, any preventive measures are expected to yield results in a span of years to decades.

Gallet et al. (2018), in drawing a parallel with adenocarcinoma of the esophagogastric junction, explain the preferential involvement of the transition zone in the olfactory cleft epithelium and the late occurrence of adenocarcinomas as related to the regression of olfactory epithelium with aging. 10 Thus, long-term follow-up of these patients is essential. 5

Barnes' histological classification does not appear to significantly impact survival outcomes. 4 5 14 Other histopathological markers may be more useful for future prognostic stratification. The literature is not consistent regarding the role of the degree of differentiation in long-term prognosis. 1 2 4 Tumor budding and the relative content of mucus may hold promise in understanding survival in these cases. 13 14

In this sample, there was a clear predominance of advanced-stage cases, which not only highlights the indolent and progressive nature of this pathology but also underscores the need to improve timely diagnosis in high-risk populations. Advanced stage is clearly associated with poorer survival outcomes. 1 2 6 More advanced neoplasms, particularly those involving critical or hard-to-access areas, lead to higher rates of intra- and postoperative complications and reduced efficacy in achieving complete resection of the disease. 7 Involvement of the brain, dura mater, and sphenoid region is thus associated with lower survival and quality of life. 13 In our study, sphenoid sinus involvement and invasion of the anterior and middle cranial fossa were not found to be negative predictors of survival.

Nodal metastases are less common than distant, as demonstrated by most authors. 1 3 5 7 13 In our sample, only one case showed regional metastasis. The low rate of nodal metastasis in adenocarcinomas, which are typically more lymphogenic, may be due to the lower density of lymphatic vessels in the sinonasal anatomy. 15 The impact on survival remains debated, but the low number of cases makes it difficult to draw definitive conclusions. 5 However, it is clear that elective neck dissection is not warranted.

Chemotherapy remains an exploratory option that may prove beneficial in advanced cases, particularly when guided by molecular studies such as TP53, mTOR and ERK analysis. 16 17 Meanwhile, the essential role of radiotherapy is well established, especially in the management of advanced-stage neoplasms. In sinonasal adenocarcinomas, adjuvant radiotherapy helps reduce the risk of local recurrence. 18 However, the relevance and role of this treatment modality must always take into consideration the selection bias, as radiotherapy is typically used in more severe cases or those with positive margins. 11 13

The extent of orbital invasion can vary, ranging from bone erosion to complete invasion of the orbital contents, including extraocular muscles and the eye itself. Traditionally, exenteration was considered necessary in cases of orbital invasion, but recent studies suggest that orbital preservation may be feasible in many cases without compromising oncologic control. A retrospective study demonstrated that 66% of sinonasal cancers with orbital invasion could be managed with orbital preservation, achieving comparable survival rates and local control to those observed in patients who underwent orbital exenteration. 19 However, when the invasion reaches the orbital apex, the situation changes significantly. Invasion of the orbital apex is recognized as an important negative prognostic factor in sinonasal adenocarcinomas. Studies have shown that the presence of invasion in this region is associated with worse clinical outcomes in terms of overall survival, local control, and distant disease control, regardless of the therapeutic approach used, highlighting the severity of this invasion pattern. 20 21 Sileo et al. (2024) emphasize that infiltration of the orbital apex is significantly correlated with an increased risk of local failure and distant metastasis, constituting an independent factor for reduced patient survival. 21 Our findings further underscore the central role of orbital apex involvement specifically in ITAC, and are consistent with the broader sinonasal cancer literature reporting worse outcomes when the apex is involved. This negative prognostic impact is largely due to the anatomical complexity of this area, which makes achieving tumor-free surgical margins nearly impossible. Involvement of the orbital apex may facilitate distant metastasis through its venous drainage into the cavernous sinus and pterygoid plexus, providing a direct hematogenous route for tumor spread independent of nodal involvement.

In the current series, the surgical option to consider was exenteration. Nevertheless, this management is still a matter of debate. Studies have shown that orbital ablation, although mutilating, can provide better outcomes in terms of local tumor control. 22 However, it is necessary to stratify and differentiate the degrees of orbital invasion. Given the negative prognostic impact of orbital apex involvement and the high rate of distant metastases in such cases, treatment decisions should consider an aggressive multimodal approach. Orbital exenteration, in combination with chemoradiotherapy, may be an option to maximize local control and attempt to delay metastatic spread.

Nevertheless, data collected from different sinonasal tumors encroaching the orbit support preservation protocols comprising of neoadjuvant chemotherapy and/or chemoradiation as preferred strategies for patients who would be candidates for orbital ablation. 23 24 In this way, it would be possible to assess the biologic response to induction chemotherapy and, thus, to serve as a biomarker to radiosensitivity. Others argue that in such advanced cases, curative treatments are not possible, and, therefore, different forms of palliative surgery or radiochemotherapy should be tailored on a case-by-case basis to enhance the quality of the patients' remaining lifetime. 20 Literature regarding ITAC orbital invasion is scarcer, so caution is advised in applying this conclusion to this tumor type as these findings are based on sinonasal cancers in general.

The limitations of the current study largely stem from its retrospective nature. The analysis of retrospective data presents inherent challenges, such as the potential absence of complete and accurate information, especially regarding certain clinical and histological variables. Some important data may be incomplete or missing, limiting the ability to perform more in-depth analyses and adequately adjust for confounding factors. Another limiting factor is the relatively small sample size. Although the study analyzed a significant number of cases, the rarity of the disease restricts the generalization of the findings. The small sample size reduces statistical power and may hinder the detection of more subtle associations between variables. Still, we consider these findings a valuable contribution as they evaluate the prognostic impact of a precise anatomical site, the orbital apex, within an ITAC restricted cohort, a distinct sinonasal malignancy with specific clinicopathologic characteristics, and clarify its association with distant metastatic spread, mortality, and oncologic management.

## Conclusion

Proactive monitoring and education in high-risk populations exposed to chronic wood dust are crucial for the early detection of ITAC, significantly improving outcomes. Orbital apex involvement is a key factor associated with poor prognosis and heightened risk of distant metastasis. Aggressive surgical approaches such as orbital exenteration must be carefully weighed against patient quality of life, with treatment decisions tailored on a case-by-case basis.
